# Evaluation of the lower extremity functional test to predict lower limb injuries in professional male footballers

**DOI:** 10.1038/s41598-024-53223-9

**Published:** 2024-01-31

**Authors:** Haniyeh Mohammadi, Raheleh Ghaffari, Abdolreza Kazemi, David G. Behm, Mahdi Hosseinzadeh

**Affiliations:** 1 Department of Sport Injuries and Corrective Exercises, Faculty of Physical Education and Sports Sciences, Shomal University, Amol, Iran; 2 Department of Physical Education, Faculty of Literature and Humanities, Vali-E-Asr University of Rafsanjan, Rafsanjan, Iran; 3 School of Human Kinetics and Recreation, Memorial University of Newfoundland, St. John’s, Newfoundland and Labrador, Canada; 4 Department of Sport Injuries and Corrective Exercises, Sport Sciences Research Institute, No. 3, 5th Alley, Miremad Street, Motahhari Street, PO Box: 1587958711, Tehran, Iran

**Keywords:** Risk factors, Population screening

## Abstract

The Lower Extremity Functional Test (LEFT) is a reliable and valid test for the measurement of athletic fitness, fatigue resistance, and speed performance. Contradictory results exist regarding the screening value of the LEFT in predicting lower limb injuries. The purpose of this study was to investigate the screening value of the LEFT in predicting lower limb injuries in professional male footballers. One hundred and twenty-one professional male football players participated in the study. LEFT was recorded pre-season and the lower-limb injuries were recorded during a 9-month season. Logistic regression analysis was used to determine the accuracy of the prognosis of LEFT. A total of twenty-five lower limb injuries were recorded. The model explained 53% of the variance in lower limb injury, showing that predictions by LEFT score is reliable, and correctly predicted 89.3% of cases, which is a large improvement. ROC analysis showed significant accuracy of the LEFT score (AUC 0.908, 95% CI 1.126–1.336, p = 0.001, OR = 1.227) in discriminating between injured and uninjured players. The optimum cut-off level of the LEFT score was 90.21 s; Our findings showed that the LEFT score was able to predict lower limb injuries in professional male footballers. The slower an athlete’s LEFT scores, the more susceptible they are to future injury risk. Sports medicine specialists, football coaches and managers are suggested to use LEFT as a pre-season screening test to identify and prevent the weakness and functional imbalance of the athletes before the injury occurs by conducting this test.

## Introduction

According to the International Football Federation, it is estimated that 270 million football players are officially registered and active worldwide^1^. The health benefits of playing football include improved cardiovascular, and musculoskeletal variables that influence the risk of life-style diseases of men^2,3^. As a high-impact sport, which has quick explosive movements, along with changes in direction, acceleration, and sudden jumps, football can induce traumatic as well as overuse injuries. These injuries may cause arthritis that occurs after anterior cruciate ligament (ACL) injury, ankle sprains limiting ankle range of motion, pain and swelling after hamstrings strain and re-injury, as well as limiting sport participation^1,4^. In football, many injury mechanisms are non-contact and mainly affect the lower limbs. Overall, injuries to this area account for 30.3–47.9% of all injuries that lead to time lost in training or competition^5^. In male footballers, the ankle and thigh are the anatomical regions most affected^6^. Also, ACL injuries have become a significant concern in athletes due to the consequences and severity of the injury^7^. The potential risk factors for injury in football include sex, age, lower limb function and morphology, anatomical and biomechanical factors, genetic factors, playing history, training, competition level, player position, dominant foot, shoes, weather, and playing field^8–10^. Some of these injury risk factors in football are non-modifiable (e.g., sex and age) and some biomechanical and neuromuscular factors are modifiable (e.g., fitness level, balance, flexibility, and strength)^1^. Neuromuscular defects cause disturbances in balance, strength, power, and create patterns that cause an increase in joint loads and compensatory movements in the lower limb^11^. However, it can be controlled through organized and focused programs^12,13^.

Interest in sports injury prevention has grown over the past 10–15 years as focused injury prevention programs reduce the rate of lower extremity injuries in athletes^13,14^. Neuromuscular, strength, technique, and balance training can prevent these injuries as they are multifactorial in nature^8,12^. Prediction and reduction of sports injuries are important for researchers and physicians. This prediction can be applied by either laboratory (e.g., isokinetic strength dynamometry)^15^ or functional movement screening tests (e.g. FMS™ test)^16^ that do not involve costly laboratory equipment. Therefore, functional screening tests can be an important part of the athletes’ evaluation process, identifying athletes that are prone to injury in order to apply injury prevention programs^17^.

The Lower Extremity Functional Test (LEFT), is a reliable and valid test for the measurement of athletic fitness, fatigue resistance, and speed by performing a series of 16 specific maneuvers as fast as possible (including forward and backward sprinting, sidestepping, cross-stepping, 45° and 90° change of direction). The test–retest reliability of the LEFT is reported to be excellent with values between 0.95 to 0.97^18,19^.

Brumitt et al.^20^ reported that pre-season LEFT scores were associated with an increased risk of lower extremity injury in 193 male and female collegiate athletes but the results of another study conducted by the same research group on 395 athletes demonstrated that LEFT is not a suitable screening tool for predicting the risk of injury in division III athletes^20,21^. Shi et al.^16^ also demonstrated the reliability and validity of the LEFT for predicting low back or lower limb injury risk in the college athletes^19^. As this test is a quick test to administer, requiring minimal personnel, and no need for special equipment, the researchers demonstrated that it warrants further consideration as pre-participatory screening examination tool for sport injury in more specific athlete populations (i.e., a more homogenous athletic population rather than heterogeneous populations like division III athletes). Considering the contradictory results obtained from previous literature and the lack of focus of previous research on football (soccer) players, and the importance of developing the screening tests in pre-seasonal athletic activity^15,16^ and physical therapy^22^, the present study aimed to investigate the probability of LEFT as a screening test to predict lower limb injuries in professional male footballers. Therefore, the purpose of this study was to investigate the screening value of the LEFT in predicting lower limb injuries in professional male footballers.

## Materials and methods

### Study design and participants

The current study was part of a prospective cohort study that investigated the prognostic ability of a battery of common pre-season functional performance screening tests to predict lower limb injuries in male athletes who are playing at the National Football League (soccer). Athletes who played in the National League One (Azadegan league) in the 2021–2022 season were eligible to participate in this study. The players were followed up as for registering their injuries for one football season (about to 9 months). A total of 121 football players participated in the study.

The study was conducted according to the principles of the Declaration of Helsinki and its latest amendments, and the protocol of the study was approved by the research ethics committees of (IR.SSRC.REC.1401.084). Registration number has been omitted due to the blinded review process. All the players signed an informed consent form to participate in the study; and each of the participant had the right to withdraw from the study at any stage of the study without any further notice.

### Injury data collection

Detailed medical information and description of lower limb injury status of each participant until the end of the competition season was recorded through monthly contacts with the participating team manager, coach, and medical staff via an online form. At the end of each month in case any injury form was not completed by any of the players, the researcher would contact the team athletic trainer to follow up on the completion of the injury registration form. All lower limb injuries (contact and non-contact) that led to a medical visit, magnetic resonance imaging, arthroscopy, or absence from training or competition were recorded.

### Lower limb injury definition

In this research, any type of lower limb injury (contact and non-contact) that kept an individual away from training or competition for 24 h, caused a visit to the doctor, or detected by magnetic resonance imaging and/or arthroscopy to be confirmed, was defined as lower limb injury^23,24^.

### Research implementation method

Before conducting the research, all the participants completed a researcher self-made personal and medical questionnaire as well as confirming their voluntary participation in the study. In a briefing session, all the players were briefed about the working method and the correct procedures to perform the LEFT test.

### Screening test

The players’ agility, speed, and resistance to the fatigue state were evaluated using the LEFT. Before starting the tests application, the players used a standard football warm-up method consisting of (5 min of running, dynamic stretching, small skips, open and close the gate, heel kicks, low shuffle and carioca)^25^.

### Lower extremity functional test (LEFT)

**Figure 1:**
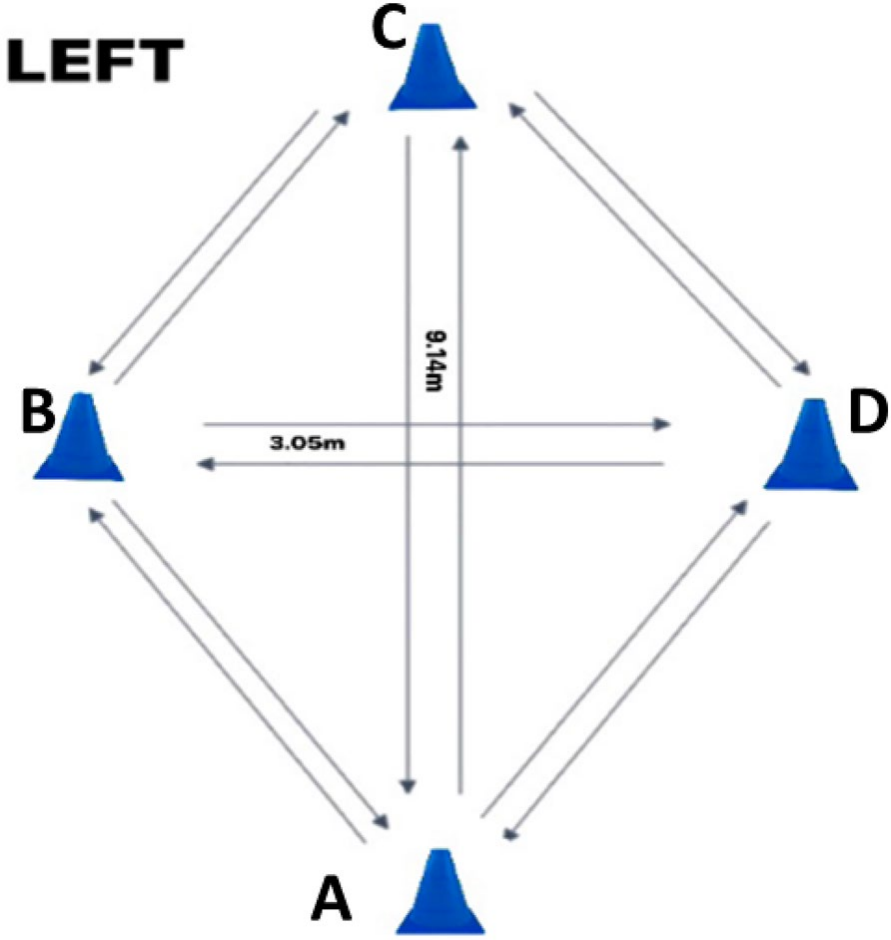
Schematic representation of the LEFT.

The LEFT test consists of 16 specific maneuvers at the maximum possible movement speed which were respectively performed without any rest interval in between (including forward and backward sprints, lateral movement, cross step, 45° and 90° cut). The test plan is a combination of four football cones (9.14 m × 3.05 m), see Fig. 1. The participants started the test standing behind the point marked by cone A. The participants performed a series of eight different agility tasks, twice (once to the right and once to the left). The maneuvers included running forward, and back, side shuffle, carioca, English figure eight, 45° cuts, 90° cuts, 90° cross cuts, running forward and backward again. Due to the multidirectional requirements of the LEFT test and the variety of tasks to be performed, verbal instruction of subsequent movements was provided during the test by the test taker, who was an expert athletic trainer. In this way, people must respond to external stimuli. If the participants failed to perform the designated maneuvers or moved the cone due to contact, the attempts were considered invalid and they had to do the test again. The running time for the first valid attempt was recorded in seconds with a stopwatch^21^. The running time started from the moment of the first sprint after the starting signal as soon as the participants left cone A to the moment of the last sprint as soon as the participants passed cone A.

### Features of the examiner

An athletic trainer with more than 3 years of experience participated in this study as an evaluator. Before the experiment, the evaluator was trained based on a structured method including instructions, setup, preparation and scoring for 2 h.

### Statistical analyses

The data is shown as mean ± standard deviation (SD) with 95% confidence intervals. The research data related to the demographic characteristics of the players and research variables were analyzed with SPSS for Windows, version 26.0, (SPSS Inc., Chicago, pernios, USA software). The p-value < 0.05 was considered significant. The Shapiro–Wilk statistical test assessed the normality of the data. Two group comparisons (injured vs. uninjured football players) were conducted using the Student *t* test. Effect sizes were calculated to compare group means with a Cohen’s d, with small effect described as values < 0.2, medium effect < 0.5 and large effects > 0.8. Logistic regression analysis was then used to create a separate model with the LEFT test score. The corresponding receiver operating curve (ROC) was plotted for defining a cut off for the LEFT performance test score.

### Ethical approval

The study was conducted in accordance with the Declaration of Helsinki, and was approved by the Ethics Committee in research of sport sciences research institute of Iran (IR.SSRC.REC.1401.084).

### Informed consent

Informed consent was obtained from all subjects involved in the study.

## Results

The total exposure time (football-sanctioned practices and competitions) in the 9-month follow-up during the 2021–2022 competition season of National League One was recorded for all athletes. Twenty-five (experiencing 23 initial injuries and 2 subsequent injuries, and only the initial injuries have been used in analysis of risk), lower limb sport injuries were recorded for 121 male footballers during the 9 month follow-ups. Age, height, weight, body mass index (BMI), and LEFT scores of all the players, are shown in Table 1. Comparisons of the LEFT performance scores showed that all the LEFT performance scores were statistically different between the injured vs. uninjured footballers (all p ˂ 0.05), (Table 1).

**Table 1:** Participant demographic data, LEFT performance score, and the results of the statistical comparisons between uninjured and injured football players.

	Participants Enrolled Total (N = 121)	Uninjured players (n = 98)	Injured players (n = 23)	p value	Effect size d [95% CI]
	Mean ± SD	Mean ± SD	Mean ± SD		
Age, (years)	22.78 ± 4.60	23.03 ± 4.65	21.70 ± 4.27	0.29	0.29 [− 0.17, 0.75]
Height, cm	180.02 ± 5.09	180.32 ± 4.82	178.78 ± 6.10	0.22	0.30 [− 0.16, 0.76]
Weight, kg	72.45 ± 6.26	72.38 ± 6.17	72.74 ± 6.77	0.50	− 0.06 [− 0.52, 0.40]
BMI, kg/m^2^	22.29 ± 1.34	22.23 ± 1.32	22.58 ± 1.41	0.492	− 0.26 [− 0.72, 0.20]
LEFT	94.06 ± 12.54	90.60 ± 9.89	108.78 ± 12.13	0.001*	− 1.76 [− 2.22, − 1.30]

*Significant differences between the uninjured and injured football players (p ˂ 0.05) in independent *t* test. *BMI* body mass index.

The separate model created by the logistic regression analysis for LEFT score is provided in Table 2. This model was statistically significant (χ^2^(1) = 48.443, p < 0.001) explaining 53% (Nagelkerke R^2^) of the variance in lower limb injury and correctly predicted 89.3% of cases.

**Table 2:** Significance of risk factor in the logistic regression model with LEFT performance.

	Coefficient	p-value	R^2^ (Nagelkerke)	Exp(B)	95% Confidence interval
LEFT	0.204	0.001	0.530	1.227	1.126–1.336

**Figure 2:**
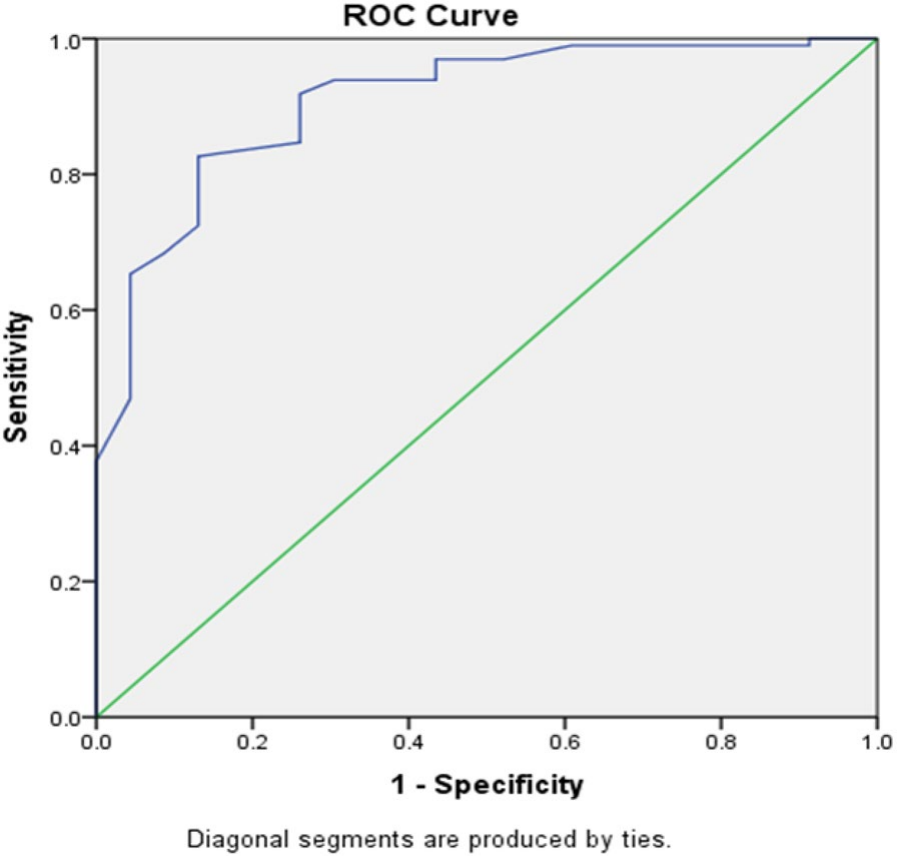
Receiver operating characteristic (ROC) curve for the LEFT performance of the footballers and lower limb injury. The straight line shows the reference line, which was approximated by the ROC curve plotted on sensitivity (true positive rate) over 1-specificity (false positive rate) for LEFT performance. Coordinates of the ROC curve shows that the LEFT performance corresponding best with the upper left hand portion of the curve was at 90.21 s demonstrating the optimal cut-point ratio for the football players participating in the study. Youden's index = (SN + SP) − 1 = (0.469 + 0.957) − 1 = 0.426.

Finally, ROC analysis (Fig. 2) showed significant accuracy of the LEFT performance score of the football players (AUC 0.908, 95% CI 1.126–1.336, p = 0.001) in discriminating between injured and uninjured players. The optimum cut-off level of the LEFT performance score of the football players was 90.21 s (where sensitivity was 0.469, and specificity was 0.957).

## Discussion

The present study investigated the ability to predict lower limb injuries in professional male footballers by LEFT. According to the results, the LEFT was able to predict lower limb injuries in professional male footballers (odd ratio = 1.227), although as a screening tool high sensitivity should be necessary, the sensitivity of LEFT in this study was 47%. In the current research, LEFT includes physical fitness factors specific to football and biomechanics of lower limb injuries. In a systematic study, Sarah de la Motte et al. investigated the relationship between physical fitness factors (flexibility, strength, speed, balance, and agility) and the risk of musculoskeletal injury finding moderate evidence for a relationship between poor balance and possible ankle injury. It also showed a moderate relationship between strength, speed, flexibility, and the risk of injury, with no significant relationship with agility^26^. Since this mentioned study was primarily conducted on a military population, many of the assessments were also specific to this population^26^. For example, in the military population, the cardiorespiratory and muscular endurance components of fitness, are more important. The present study was conducted however on a large population of football/soccer players. In the football population, balance, strength, speed, and agility are also of important components of physical fitness and the screening tests were selected for this reason.

Since football is associated with cutting movements, jumping, changing direction in the shortest possible time, the test considered in this research is compatible with football techniques. The use of reliable functional screening tests is one of the important challenges of researchers. Sinicarelli et al. investigated test–retest reliability in a set of functional tests. They conducted seven tests on 22 adults (14 men, 8 women) in 2 sessions and 7 days apart. The tests included Y-Balance Test, Single-Leg Countermovement Jump Test, Single-Leg Hop for Distance Test, Side Hop Test, Speedy Jump Test, Lower Extremity Functional Test, Agility *T*-Test. The results showed the high reliability of the functional test set for potential use in clinical sports training^27^. The LEFT was used in past literature to assess athletic readiness to return to sports after a traumatic knee injury^28^, according to which men had to complete this test for 100 s (range 125–90). But in this study, we used LEFT as a functional screening test to predict lower limb injury in professional male soccer players, according to the findings, this test is a reliable predictor of lower limb injury in male professional football players, and also a cut-off point of 90.21 indicates that the slower an athlete’s LEFT scores, the more susceptible they are to future injury risk. In a similar study, Brumitt et al. investigated LEFT and Lower Quadrant Injury in 189 NCAA Division III college athletes (106 women, 83 men) from 15 teams. According to their findings, male athletes completed LEFT faster (105 ± 9 s) than their female counterparts (117 ± 10 s). Furthermore, female athletes who did 3–5 h of plyometric exercises per week had a better score than female athletes who did less than 3 h of plyometric exercises. Moreover, female athletes with slower LEFT scores were more prone to injury, but there was no correlation between LEFT scores and the level of injury in male athletes^29^. This study is generally consistent with our study, but inconsistent considering sex, which may be due to the heterogeneity of the sample. Also, according to our findings, 25 injuries were reported during one season, 23 initial injuries and 2 subsequent injuries, muscular injuries had the highest incidence (36%), with groin strains the highest occurrence. After muscle injuries, ligament, and knee injuries accounted for the next highest incidence (32%). In most studies, the knee, especially the anterior cruciate ligament, are the most severe injuries that occur through both contact and non-contact mechanisms^30^. In our study, this result was consistent in terms of time loss. After that, the ankle was the most frequently injured (28%). One of the reasons for injuries in this area is due to the pivotal role of this joint and it is the joint closest to the ball. Therefore, dribbling, tackling, and shooting happen more often in this area^31,32^.

### Research limitations

In this research the daily activities, diet and recovery of each player were not controlled individually as well the mental state of the players was not controlled. More prospective cohort studies are necessary to confirm the predictive utility of LEFT in homogeneous sports populations or as one of the components of a battery of pre-season performance tests. This study should also be conducted on female footballers as well. Also, exposure time and previous injury affect the amount of injuries of athletes, however the athletes and coaches involved in this study did not give us the permission to use this information, so classification should also be done in this case. Finally, the running time was recorded in seconds with a stopwatch while using photocells would be more reliable.

## Conclusion

The present study showed that slower scores in LEFT have a significant relationship with future injuries in the lower limb of professional male footballers, so sports medicine specialists and athletic trainers can consider using the LEFT as a reliable screening test to predict the professional male footballers’ probability to sustain lower limb injury.

### Applied, clinical relevance

Since LEFT was able to predict the injuries of the lower limb of male professional footballers, sports medicine specialists, team coaches, professional football league officials and clubs are suggested to use this test to identify and prevent the weakness and functional imbalance of the athletes before the injury occurs by conducting this test (especially in the basic level). Furthermore, during the pre-season evaluations and before the start of the contract with the player in question, the football clubs (teams) managers can use screening tests to evaluate the players in terms of the risk of sustaining sports injuries. It can also be said that the final results of the current research can reduce the economic and medical burden to teams caused by the injuries and the personal health of the footballers and ultimately increase the level of health and quality of the football community.

## Data Availability

The datasets used and analyzed during the current study are available from the corresponding author, MH, upon reasonable request.
